# Water Absorption and Solubility of Fluoride-Based Restorative Materials Exposed to Ionizing Radiation

**DOI:** 10.3390/polym17202736

**Published:** 2025-10-13

**Authors:** Sara Čekalović Agović, Eva Klarić, Ana Ivanišević, Majana Soče, Timor Grego, Irena Radin Nujić

**Affiliations:** 1 Department of Endodontics and Restorative Dentistry, School of Dental Medicine, University of Zagreb, 10000 Zagreb, Croatia; sara.cekalovic@gmail.com (S.Č.A.); aivanisevic@sfzg.hr (A.I.); irena.radin@gmail.com (I.R.N.); 2 Radiotherapy Unit, Department of Oncology, University Hospital Centre Zagreb, 10000 Zagreb, Croatia; majana.soce@gmail.com; 3 UPMC Hillman Cancer Center Croatia, 49210 Zabok, Croatia; timorgrego@gmail.com

**Keywords:** head and neck radiotherapy, fluoride-releasing restorative materials, water absorption, solubility

## Abstract

Background: Radiotherapy is a key treatment for head and neck cancers but often compromises oral health, partly through its impact on restorative materials. A specific concern is whether ionizing radiation alters the water absorption and solubility of fluoride-releasing restoratives, potentially affecting their durability. This study aimed to evaluate these properties following clinically relevant radiation exposure. Methods: Seven contemporary fluoride-releasing restorative materials (Fuji IX, Equia Forte HT, Fuji Triage, Activa Presto, Cention, Luminos, and Beautifil II) were tested (n = 10 per group). Specimens were either irradiated with 70 Gy in 35 fractions using a clinical linear accelerator or maintained as non-irradiated controls. Water absorption and solubility were measured over 35 days according to ISO 4049 protocols, and data were analyzed with repeated-measures ANOVA. Results: Across all materials, irradiated specimens exhibited slightly higher water absorption and solubility values compared to controls; however, differences were not statistically significant (*p* > 0.05). Material-specific trends were observed, with Fuji IX, Fuji Triage, Beautifil II, and Equia Forte showing relatively higher absolute values. Conclusions: Clinically relevant ionizing radiation did not significantly affect the water absorption or solubility of the tested fluoride-releasing restorative materials, suggesting preserved physicochemical stability under therapeutic conditions.

## 1. Introduction

In 2020, approximately 930,000 cases of head and neck cancer were diagnosed worldwide, with radiotherapy representing a cornerstone of treatment. Despite advances in radiation techniques, nearly all patients experience adverse effects such as mucositis, dysgeusia, and, most notably, long-term hyposalivation [1]. Since radiotherapy is the primary treatment for head and neck cancer (HNC), side effects could affect oral tissues like salivary glands, oral mucosa, and dental structures [2], which would be detrimental to this population’s health and quality of life. Significant morphological alterations in dentin have been observed during radiotherapy [3], which could jeopardize adhesive restorations made after the cancer treatment period [4,5]. Although international consensus exists regarding caries prevention, the effectiveness of these preventive strategies in patients treated with head and neck radiotherapy remains uncertain [6]. Furthermore, limited data and a lack of specific guidelines hinder supportive care in this population [7,8]. The structural integrity of hard tooth tissues is also negatively impacted by radiotherapy. The crystalline structure of enamel and dentin can be altered by ionizing radiation, which can result in demineralization and the development of microcracks, which are characterized by a decrease in the calcium/phosphorus ratio. As a result, these modifications reduce the dental tissues’ overall biomechanical qualities, permeability, and surface hardness [3,9]. A particular challenge concerns the iatrogenic exposure of dental tissues and restorative materials to radiation. High-energy therapeutic radiation has been shown to negatively affect both teeth and restorative materials [10,11]. Clinical evidence indicates that patients undergoing head and neck radiotherapy experience shortened restoration longevity [12]. However, it remains unclear whether these failures are primarily attributable to direct radiation effects on hard dental tissues and restorative materials, or to secondary consequences such as alterations in the oral microbiome and salivary reduction [13,14]. While numerous studies have examined radiation effects, the findings are inconsistent. Reported outcomes range from improved properties, such as increased tensile strength and microhardness of resin-modified glass ionomers, to deleterious effects, including reduced flexural strength of glass ionomers and decreased microhardness of resin composites [15,16,17,18,19,20,21]. Such heterogeneity is likely due to variations in material composition, structural characteristics, and radiation protocols, including single-dose versus fractionated regimens.

Composite restorative materials consist of an organic polymer matrix, a bonding interface, and dispersed inorganic fillers. The organic matrix, first introduced by Bowen, is typically based on high-molecular-weight dimethacrylates (DMA). Modern composites often employ bisphenol-A glycidyl methacrylate (Bis-GMA), a highly viscous resin commonly blended with lower-viscosity monomers such as triethylene glycol dimethacrylate (TEGDMA) to optimize handling [22,23]. Inorganic fillers—including quartz, silica, borosilicate glass, and aluminosilicates of lithium, tin, strontium, and zirconium—are incorporated to enhance mechanical and chemical performance. Filler-matrix adhesion is generally achieved using silane coupling agents, though more recent approaches employ covalent bonding strategies [24,25]. Clinically, composites are categorized by filler size and volume fraction into microfilled, hybrid, microhybrid, flowable, and packable subgroups.

Compared to microfilled composites, hybrid composites demonstrate a higher modulus of elasticity, reduced polymerization shrinkage, greater degree of conversion, and improved resistance to water absorption and biodegradation, all of which enhance restoration quality [23,26]. Increasingly, emphasis has been placed on bioactivity—defined as the ability of a material to promote remineralization and strengthen adhesion to dental tissues through ion release. Such bioactivity is closely linked to the interaction between restorative materials and dentin substrates, whether or not demineralization processes are present [26].

Resin-based composites, known for their favorable mechanical and aesthetic properties, have been further enhanced by fluoride incorporation, giving rise to a range of hybrid products. For instance, Luminos UN (Unodent, Witham, UK) is a nanohybrid composite containing fluoride, based on a Bis-GMA/TEGDMA resin matrix with fluoroboroaluminosilicate fillers [22,23,26,27]. Activa Presto (Watertown, Pulpdent, MA, USA) is a bioactive light-cured composite comprising diurethanes, methacrylates, modified polyacrylic acid, silica, amorphous calcium phosphate, and sodium fluoride. This material is designed to release ions, stimulate remineralization, and recharge from saliva or dietary sources [27]. Such bioactive composites, in addition to fulfilling conventional restorative requirements, promote remineralization of dental tissues and cavity margins by releasing calcium, phosphate, and fluoride ions, and are increasingly considered the preferred option for caries management [27].

More recently, alkasite restoratives have been introduced, including Cention N and the next-generation Cention (Ivoclar Vivadent, Schaan, Liechtenstein). These encapsulated materials contain both organic and inorganic components. The organic matrix comprises four dimethacrylates—UDMA, DCP, aromatic-aliphatic UDMA, and PEG-400 DMA—providing mechanical strength and hydrophilicity for effective wetting of dental tissues. The inorganic fillers include barium–aluminum–silicate glass, ytterbium trifluoride, Isofiller, and calcium-fluorosilicate glasses. Clinically, Cention offers the advantage of dual curing (light polymerization and acid–base reaction) and can be applied with or without an adhesive system. Its inorganic fillers not only improve strength but also enable sustained release of fluoride, calcium, and hydroxyl ions, contributing to remineralization and caries prevention [28]. Recent studies indicate that Cention exhibits a greater capacity for long-term fluoride release compared to other fluoride-releasing composites such as Luminos UN and Activa [29,30,31].

Although fluoride is beneficial for preventing dental cavities, possible environmental contamination must also be taken into account. Fluoride is a pollutant of the environment (causing toxicity and fluorosis at greater quantities) and a public health agent (preventing caries at low amounts). Deliberate fluoridation and regulated fluoride distribution (for example, through dental materials and public water systems) and defluoridation and recovery from groundwater and industrial effluents are the two parallel paths that research and technical activities have taken as a result. In order to meet more stringent water-quality requirements and to facilitate resource recovery, various modern defluoridation technologies have been developed or optimized during the past five years. These technologies vary from well-established chemical and adsorption techniques to sophisticated membrane, electrochemical, and hybrid recovery systems [32].

Recent in vitro work suggests that therapeutic ionizing radiation can measurably alter the water transport behavior of fluoride-releasing restorative materials, but effects are material- and protocol-dependent. The water sorption and solubility of resin-based materials were determined according to ISO 4049:2019—Dentistry—Polymer-based restorative materials (International Organization for Standardization, 2019) [33]. Under single or cumulative high doses typical of head-and-neck radiotherapy (≈30–60 Gy), several studies report significant increases in ISO-4049 water sorption and solubility for resin composites and glass-ionomer–based materials, indicating radiation-induced network changes and leachable loss of components (e.g., plasticizers/fillers) [34]. Baseline differences remain important: long-term assessments without irradiation show that conventional and resin-modified glass ionomers generally exhibit higher sorption than resin composites, and alkasite materials such as Cention may exceed ISO solubility limits in self-cure but perform better when light-cured—highlighting composition and curing mode as key determinants of water uptake and mass loss. Radiation can also increase surface roughness and wettability of resin composites and RMGICs, plausibly promoting additional water ingress and subsequent solubility [35,36]. By contrast, some studies using fractionated, clinically relevant regimens (2 Gy × 35 to 70 Gy) observed minimal adverse changes in selected fluoride-releasing materials’ mechanical/surface properties, underscoring that dose delivery and storage media strongly mediate outcomes; direct evidence on sorption/solubility under fractionation remains limited [35]. Methodology also drives variability: ISO 4049 defines sorption/solubility thresholds and specimen protocols, and recent work shows that specimen dimensions and immersion conditions can meaningfully shift measured values [37]. Finally, irradiation has been shown to enhance fluoride release from glass ionomers—an effect likely intertwined with water diffusion pathways—supporting their continued use while reinforcing the need for standardized sorption/solubility testing under clinically realistic irradiation [38].

### Study Aim

The present study aimed to evaluate the water absorption and solubility of fluoride-releasing restorative materials following exposure to ionizing radiation. The working hypothesis was that radiotherapy, as employed in the management of head and neck malignancies, does not significantly alter these material properties.

## 2. Materials and Methods

### 2.1. Sample Size Determination

The sample size for planned comparisons was calculated using the G*Power program (version 3.1.9.7; Heinrich-Heine-Universität Düsseldorf, Düsseldorf, Germany) based on the effect size (Cohen’s f = 0.40), with a significance level set at 0.05 and a power level of 0.8 (high performance size), and a minimum required sample size of 10 per group was calculated.

### 2.2. Sample Preparation

Seven contemporary fluoride-releasing dental restorative materials were evaluated, with ten specimens prepared per group (n = 10). The materials included Fuji IX (GC, Japan), Equia Forte HT (GC, Tokyo Japan), Fuji Triage (GC, Tokyo, Japan), Activa Presto (Watertown, Pulpdent, MA, USA), Cention Forte (Ivoclar, Schaan, Liechtenstein), Luminos (Unodent, Witham, UK), and Beautifil II (Sofu, Kyoto, Japan). For clarity, the materials are abbreviated as follows: Fuji IX (F9), Equia Forte HT (EQ), Fuji Triage (F3), Activa Presto (AP), Cention (C), Luminos (L), and Beautifil II (BF). The compositions and relevant characteristics of the tested materials are summarized in Table 1. Specimens for fluoride release and reabsorption tests were prepared using Teflon molds measuring 6 × 4 mm, while 8 × 2 mm molds were used for water absorption and solubility tests. To minimize air entrapment, polyester strips were placed on both sides of the molds, and the materials were pressed with a glass slide. Encapsulated materials (F9, EQ, F3, and C) were mixed according to the manufacturers’ instructions using an automatic capsule mixer (Silver Mix, GC Corporation, Tokyo, Japan). Light-curing materials (AP, L, C, and BF) were polymerized for 20 s using a Bluephase Style lamp (Ivoclar Vivadent, Schaan, Liechtenstein) at 1200 mW/cm^2^, with curing intensity verified using a Bluephase radiometer Meter II (Ivoclar Vivadent, Schaan, Liechtenstein). All specimens were polished sequentially using 4000-grit silicon carbide paper (Buehler, Düsseldorf, Germany) followed by silicon polishing pastes of 0.1, 0.3, and 0.05 µm particle size, employing a polishing machine (Minitech 250, Presi, Évans, France). Polished specimens were rinsed with distilled water and stored in polyethylene Eppendorf tubes (Kefo, Sisak, Croatia) containing 5 mL of deionized water at 37 °C (INEL, Zagreb, Croatia) until testing. Between measurements, water was refreshed daily.

Specimens were divided into two groups: control (non-irradiated) and experimental (irradiated) (n = 10 per group). Experimental specimens were exposed to ionizing radiation at the Department of Head and Neck Radiotherapy, KBC Zagreb, receiving a total dose of 70 Gy over 35 days (2 Gy per day), simulating the standard therapeutic regimen for head and neck cancer. Irradiation was performed using a Siemens Primus linear accelerator (Siemens Healthineers AG, Erlangen, Germany) with a 6 MV photon beam at a source-to-surface distance of 100 cm. One Gray (Gy), the SI unit of absorbed dose, is equivalent to 100 rad and represents the absorption of 1 joule of energy per kilogram of material (1 J/kg); thus, 0.01 Gy corresponds to 100 ergs per gram. Between irradiation sessions, specimens were maintained in deionized water at 37 °C, with daily water replacement.

### 2.3. Water Absorption and Solubility

**Figure 1:**
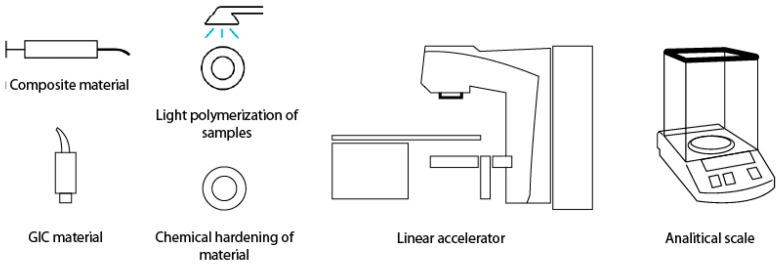
Sample preparation, irradiation procedure and mass measurement.

Following irradiation, all specimens—including control samples stored in distilled water—were placed in a desiccator containing freshly dried silica gel. Samples were weighed daily using an analytical balance (MS105 NewClassic, Mettler-Toledo AG, Nänikon, Switzerland) until the mass change between consecutive measurements was less than 0.1 mg, establishing the initial dry mass (m_1_). Subsequently, each specimen was immersed in 4 mL of distilled water within conical-bottom plastic containers (Eppendorf tubes) and stored in the dark in an incubator at 37 ± 1 °C. Mass measurements were conducted at 1, 7, 14, 21, and 35 days of immersion (m_2_(t), where t represents the immersion time). Prior to weighing, samples were removed from the water, gently blotted to remove surface moisture, and air-dried for 15 s. The distilled water was refreshed every seven days (Figure 1).

After the immersion period, specimens were returned to the desiccator and weighed daily until a constant mass was achieved (mass difference <0.1 mg), representing the final mass (m_3_). Consistent with the manufacturer’s instructions, the analytical balance was zeroed before each measurement, and weights were recorded only after stabilization of the balance reading. Each sample was measured in triplicate, and the mean value was used for calculations. Water absorption and solubility were calculated in absolute terms (µg/mm^3^) using the equations:

Water absorption: WA = (m_2(eq)_ − m_3_)/V

Solubility: S = (m_1_ − m_3_)/V

where m_2(eq)_ denotes the mass at equilibrium and V is the specimen volume.

### 2.4. Statistical Analysis

Data are presented in tables and figures. Due to the non-normal distribution of fluoride measurements, descriptive statistics are reported as median and interquartile range. Bond strength distributions for each group are illustrated using box plots. The effects of treatment were assessed using a multifactorial repeated-measures ANOVA, with material type, group (control or experimental), and time point (1–35 days) as factors. Levene’s test indicated heterogeneity of variance among materials (*p* < 0.001); therefore, the ANOVA model allowed separate variances for each material. The covariance structure was selected to minimize the Akaike Information Criterion (AIC), resulting in a first-order heterogeneous autoregressive model, which accounts for decreasing correlation between measurements as time intervals increase. Statistical analyses were conducted using SAS software, version 9.4 (SAS Institute Inc., Cary, NC, USA).

## 3. Results

**Figure 2:**
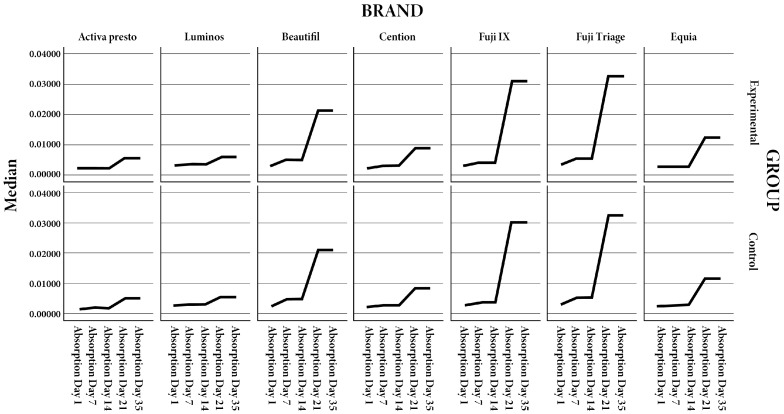
Presentation of the mean value of absorption by day, material and group.

Water absorption and solubility were assessed for all tested fluoride-releasing restorative materials over 35 days. For most materials, irradiated specimens exhibited slightly higher water absorption compared to controls; however, these differences were not statistically significant (*p* > 0.05) (Figure 2 and Table 2). Activa Presto, Luminos, and Beautifil II showed a gradual increase in median absorption over time, with Beautifil II displaying a more pronounced rise after 21 days, though intergroup differences remained non-significant. Cention consistently maintained low absorption values throughout the study period. Fuji IX and Fuji Triage demonstrated greater absorption at 21 and 35 days, yet differences between irradiated and control groups did not reach statistical significance. Equia Forte showed minor temporal variability without statistically significant intergroup differences. Regarding solubility, irradiated specimens of all materials exhibited marginally higher values than their respective controls; however, no statistically significant differences were observed at any time point. Overall, these findings indicate that exposure to clinically relevant ionizing radiation did not significantly affect the water absorption or solubility of the tested fluoride-releasing restorative materials.

## 4. Discussion

Dental restorative materials are exposed to harmful environmental influences over time in the oral cavity. Continuous contact with saliva can cause their dissociation, release of soluble substances and corrosion, especially in the presence of acids. These chemical processes lead to a decrease in the mechanical strength, stability and life of the materials themselves. In addition, the release of constituent elements into the oral environment can negatively affect the biocompatibility of these materials. Water absorption and solubility are among the chemical properties of dental materials, and most of them show some degree of sensitivity to these processes [32].

Ionizing radiation has a short wavelength and high energy, and interacts with dental materials and dental tissues via electrostatic and electromagnetic forces [39]. The properties of restorative materials can change proportionally to the increase in the amount of ionizing radiation. Although the effect of ionizing radiation delivered during radiotherapy procedures on restorative materials has not been fully investigated [32]. According to the literature, composite materials have the lowest water absorption ability compared to other restorative materials—they absorb only 0.17% water. They are followed by compomers with 1.1%, while resin-modified glass ionomer cements showed the highest absorption level, as much as 6%, which are also the weakest results [32]. Such results are influenced by the different composition and hydrophilicity of certain parts of the mentioned dental restorative materials. Glass ionomers are hydrophilic materials and absorbed water has a major factor in the acid-base reaction of hardening [32]. Some of the negative consequences of absorbed water on materials are discoloration and changes in the mechanical properties of the material, while the concentration of absorbed water and the solubility of the composite are determined by the type and concentration of the composite resin, the amount of filler, and the degree of conversion of monomer into polymer. It has been proven that the organic matrix is responsible for the diffusion-regulated process of water absorption, while the filler components are responsible for the solubility of the material [40,41]. Previous studies of composite materials have proven the connection between the components of composite materials and the possibility of water absorption. A prominent negative correlation has been demonstrated for materials with a lower filler content. Since absorption is a characteristic related to the polymer phase, an increased filler content causes a reduced proportion of the polymer matrix and thus lower water absorption [41]. The Cention alkali composite contains filler particles that make up 78.4% of the weight of the material. The stated values can be an assumption that the aforementioned materials will have lower water absorption, as in Active Presto and Luminosa. In addition to the monomer content, their characteristics also affect the amount of liquid absorption. Hydrophobic resins had lower water absorption compared to hydrophilic ones. The Cention alkalite composite has in its structure monomers: aromatic aliphatic UDMA, DCP and PEG-400 DMA. All of the above, excluding PEG-400 DMA, are hydrophobic monomers and it can be concluded that hydrophobic monomers form a larger proportion, although this is not stated by the manufacturer. Although solubility and water absorption do not always occur to the same extent, Alshali et al. have proven the connection between absorption and solubility of materials in their research [41].

The solubility of the composite is explained by the presence of residual monomer, filler and different additives. The concentration of residual monomer in the highest concentration depends on the degree conversions and about the type of monomer found in a certain material. By increasing the degree conversion, the amount of residual monomer decreases and the solubility of the restorative increases material. It can be concluded that materials based on composites (Luminos, Activa Presto and Cention) in this study did not show statistically significant water absorption and neither solubility neither in the experimental nor the control group. Beautfil is a giomer hybrid material that has the possibility of releasing fluoride, and for this process it needs to absorb a certain amount of water in order for the process of diffusion and release of ions to occur [42]. In the study by Gonulol et al. compared is the water absorption and solubility of the giomer composite and the two nanohybrid composite resins. Disk-shaped samples were made of giomer (Beautifil II, Shofu, Kyoto, Japan) and two nanohybrid composites. Water absorption and solubility were assessed by weight gain or loss after 28 days of storage in water. Water absorption (%) varied among groups, with Beautifil II showing the highest values (*p* < 0.001). No statistically significant differences in water solubility values were observed among groups (*p* = 0.66), which is similar to the results of this study where Beautifil showed higher water absorption in both groups over a 35-day period compared to the composite materials Luminos, Activu Presto, and Cention. Their explanation is that the hydrophilicity of the monomers had an effect on the higher absorption results. Beautifil does not contain UDMA, which is more hydrophobic compared to Bis-GMA and TEGDMA found in the aforementioned material. We can conclude that a high level of water absorption can have a negative effect on cavity restoration.

The results of this study did not show a statistically significant difference, which could be a consequence of the above-mentioned effect of the filler content on fluid absorption. Glass ionomers have poorer mechanical properties compared to composite materials due to the hardening reaction and water sensitivity, and in order to prevent the weakening of mechanical properties, coatings are used that reduce dehydration and water absorption of the cement [43,44]. Since the samples used in this study were not coated, the results obtained can be explained by the fact that the highest absorption and solubility in both subgroups of materials was in the following order: Fuji IX > Fuji Triage > Beautifil > Equia > Cention > Luminos > Activa Presto. In the study by Savas et al., the aim was to investigate the effects of different beverages on water absorption and solubility in restorative materials based on glass ionomer cement. After 28 days, Glasiozit showed the lowest water absorption (16.75 μg/mm^3^) of the tested materials, while Ketac N100 (155.41 μg/mm^3^) and GCP Glass Fill (161.01 μg/mm^3^) had the highest water absorption [45], suggesting that the composition of restorative materials plays a key role in their water absorption and solubility. The results of the study by Jih et al. provide important insight into the hydrophilic properties of alkasite materials, specifically Cention N, compared to other restorative materials such as conventional composites, giomers, and glass ionomer cements. Alkasite materials represent a new generation of restorative materials with potential bioactive properties, but their behavior in the oral environment, especially in the context of water absorption and solubility, requires additional research. In a study by Jih et al., Cention N showed significantly higher water absorption compared to conventional composite resins but lower compared to glass ionomer cements [46]. This can be attributed to its alkaline, ion-releasing matrix, which contains glass fillers responsible for the release of hydroxyl and fluoride ions. Despite the increased absorption, the solubility of Cention N was within acceptable limits, indicating that the material can maintain its dimensional stability and mechanical properties under clinical conditions. As previously documented in the literature, high water absorption of restorative materials on the one hand allows for ion diffusion and creates remineralization conditions, while on the other hand it can have a negative effect on the mechanical properties of the material and lead to degradation of the resin matrix and compromise the margins of cavity fillings. However, Cention N did not show significant degradation in this study, making it acceptable for clinical use as a restorative material and the material of choice in patients with high caries risk. It is important to note that the results also significantly depend on the length of exposure time to the aqueous medium, which further confirms the importance of long-term testing of dental restorative materials. Additionally, the fact that all tested materials showed some level of absorption and solubility emphasizes the need for careful selection of materials based on the clinical requirements of specific patient situations and cases. Based on this study, it can be concluded that the alkali-based restorative material Cention N exhibits higher water absorption compared to conventional composites, but lower compared to glass ionomer materials [46]. The results obtained indicate the need for careful selection of dental materials depending on the requirements of the clinical situation—especially in cases where long-term dimensional stability and minimal migration of substances from the material are important, and in patients at high risk for caries, such as patients undergoing head and neck radiotherapy. Although increased absorption may allow for ion exchange and bioactivity, potential degradation of the organic matrix of the material remains a challenge in the long term [46].

In a study by de Amorim et al., it was demonstrated that ionizing radiation alters the surface properties of dental materials, especially glass ionomers. Glass ionomer materials are more susceptible to degradation, with increased hydrophilicity and reduced microhardness [35]. However, the present study did not confirm such pronounced changes, which may be explained by differences in irradiation protocols. Many previous investigations, including that of de Amorim et al., employed single high-dose irradiation to simulate cumulative exposure, thereby producing acute matrix disruption and exaggerated degradation effects. In contrast, this study applied a fractionated protocol that more closely reflects clinical radiotherapy regimens (e.g., daily low doses over several weeks). Fractionated delivery allows intervals between exposures, during which restorative materials may undergo partial recovery processes, such as rehydration, radical recombination, or structural reorganization. This temporal buffering likely mitigated the cumulative physicochemical damage, resulting in lower water absorption and solubility values. These methodological differences highlight the importance of radiation dosing strategy in explaining heterogeneity across studies and suggest that fractionated protocols may provide more clinically relevant insights. Composite materials exhibit more stable behavior and may be more resistant under radiotherapy conditions. They demonstrated that radiation leads to an increase in the hydrophilicity of the surface of dental materials by measuring a decrease in the contact angle, which favors greater penetration of water into the structure of the material itself. Radiation can also cause degradation of the organic matrix (in composites), breakdown of ionic bonds (in glass ionomers), and disruption of structural homogeneity. These changes have a negative impact on the properties of the material and result in increased water absorption, reduced microhardness, increased surface roughness, and increased solubility of the material [35].

Water absorption and solubility are key physicochemical parameters in assessing the long-term stability and clinical efficacy of dental restorative materials. Their importance is particularly evident in situations where the oral environment is compromised, such as in patients undergoing head and neck radiotherapy. Ionizing radiation, which is used for therapeutic purposes, can induce a series of structural and chemical changes in dental materials, which directly or indirectly affect their resistance to aqueous media and dissolution. Numerous studies, especially those conducted in vitro, have shown that materials containing hydrophilic components such as conventional and glass ionomer cements, as well as bioactive composites, show a greater tendency to absorb water. Due to their ionically crosslinked matrix and affinity for polar solvents, glass ionomer materials absorb water in significant quantities, which can lead to swelling, loss of mechanical strength and increased solubility. Although some studies have shown that ionizing radiation increases the surface roughness and hydrophilicity of resin-based composites, these effects do not always translate into measurable differences in water absorption or solubility. One possible explanation for this discrepancy is that surface roughness reflects primarily superficial matrix degradation and microcrack formation, while water absorption and solubility are governed by deeper properties such as filler loading, resin crosslinking density, and hydrophobic/hydrophilic monomer balance. In our study, materials such as Cention, Activa Presto, and Luminos possessed high filler content and predominantly hydrophobic monomers, which likely stabilized their internal network against water diffusion despite potential minor surface alterations. Furthermore, the use of a fractionated radiation protocol, as opposed to single high-dose exposure, may have minimized acute matrix disruption and allowed for structural recovery between doses. Together, these factors help to explain why radiation did not significantly alter water absorption or solubility in the present investigation, even though microstructural surface changes have been observed in other studies.

Among the materials tested, giomers (Beautifil II) and alkasites (e.g., Cention) showed different degrees of sensitivity to radiation. Giomers, due to their complex structure containing S-PRG, have a pronounced hydrophilicity and a tendency to change under the influence of radiation, while alkasites like Cention show better stability. Bioactive composites Activa Presto, due to their resinous component and adaptive behavior, show relative resistance to the degradation effects of radiation. What further complicates the interpretation of the results is the significant variability in the testing methodology: different radiation doses, different sample preparation protocols, variety of materials, as well as storage conditions (dry/wet environment). However, there is increasing evidence confirming that radiation exposure—especially at therapeutic doses of 60–70 Gy—can have a cumulative effect on the degradation of dental materials, especially in aspects related to water (absorption, solubility, ion release) [18,35]. In view of the above, in the clinical context caution is recommended when choosing materials for patients undergoing head and neck radiotherapy. Lower grade materials hydrophilicity, better stability and higher resistance to radiation (e.g., modern bulk-fill composites and certain alkasites) can provide longer-lasting results and a lower risk of post-therapy complications.

Further research is needed to standardize protocols for testing water absorption and solubility under conditions that simulate radiation, as well as longitudinal clinical studies to confirm in vitro results in real-world oral conditions.

Since there are no similar studies in the available literature, reference was made to studies that generally dealt with the absorption and solubility of dental restorative materials, the results of which support the stability of the material itself. Ionizing radiation did not exert a positive effect on water absorption or solubility in any of the tested materials. Furthermore, in our previous study conducted by the same group of investigators, no statistically significant negative effects of a therapeutic dose of radiotherapy on the mechanical or dimensional stability of alkasite and glass hybrid materials were observed. Only a minor negative effect, limited to discoloration, was detected in the conventional resin composite used as a reference material [35]. The materials that showed a significant level of fluoride release and uptake, and a low level of absorption and solubility over a period of 35 days are Cention and Equia Forte, which supports the stability of the material, which is important for us when making fillings, and the release and re-uptake of fluoride provides us with anti-cariogenic conditions in patients undergoing head and neck radiotherapy who are considered to be at high risk of caries. It is important to take into account the concentrations of the evaluated bioactive materials, as well as environmental factors and the experimental conditions used. In addition, this work only investigated the release and uptake of fluoride, although other ions, especially calcium ions, are equally important in the remineralization process. Further research will enable scientists to learn more about the processes underlying fluoride release from these dental materials, as well as their long-term release patterns and potential advantages, such as long-term benefits against carcinogenesis. In addition, investigation of different coating processes or modifications to enhance fluoride release from EQUIA Forte HT and other modern restorative materials could lead to the development of more effective preventive dental measures. It would also be useful to investigate the relationship between the concentrations of fluoride ions released and actual clinical outcomes in caries prevention. Assessing the effectiveness of these materials in practical situations and evaluating their clinical effectiveness over time could provide a more thorough understanding of their utility and help clinicians select the best materials for their patients.

The findings of this study indicate that fluoride-releasing restorative materials, including composites, giomers, and alkasites, maintain stable water absorption and solubility following exposure to therapeutic doses of ionizing radiation. Materials such as Cention N and Equia Forte, which demonstrated significant fluoride release and re-uptake while maintaining low absorption and solubility, appear particularly suitable for use in patients undergoing head and neck radiotherapy, who are at elevated risk for dental caries. The hypothesis that radiotherapy, as employed in the management of head and neck malignancies, does not significantly alter these material properties was therefore accepted.

The combination of dimensional stability and bioactive ion release supports both restorative longevity and anti-cariogenic efficacy. Clinicians should consider material composition, hydrophilicity, and filler content when selecting restoratives for high-risk patients, prioritizing those with low water absorption, low solubility, and proven fluoride release. Further longitudinal and in vivo studies are warranted to confirm these in vitro findings and to optimize preventive dental strategies in the context of radiotherapy. The findings of this study indicate that fluoride-releasing restorative materials, including composites, giomers, and alkasites, maintain stable water absorption and solubility following exposure to therapeutic doses of ionizing radiation. Although other investigations have demonstrated radiation-induced surface roughness or increased hydrophilicity, our results suggest that such superficial alterations do not necessarily compromise the physicochemical stability of the material. This distinction is clinically important: dimensional stability and controlled ion release—rather than minor surface textural changes—are more relevant to long-term restorative performance in irradiated patients. Materials such as Cention N and Equia Forte, which demonstrated significant fluoride release and re-uptake while maintaining low absorption and solubility, appear particularly suitable for use in patients undergoing head and neck radiotherapy, who are at elevated risk for dental caries.

### Limitations of the Study

Despite providing valuable insights into the water absorption, solubility, and fluoride release of restorative materials under ionizing radiation, this study has several limitations. First, the investigation was conducted entirely in vitro, and the oral environment is far more complex, with fluctuating pH, temperature variations, mechanical forces, and enzymatic activity, all of which can influence material behavior and may alter the observed results. Second, only a limited number of restorative materials were tested, which may not fully represent the wide range of fluoride-releasing materials available on the market. Third, the radiation protocol, while designed to simulate therapeutic doses for head and neck cancer patients, may not account for individual variations in clinical radiotherapy procedures, including differences in fractionation, beam orientation, and tissue interactions. Fourth, the study focused exclusively on water absorption, solubility, and fluoride release, without assessing other relevant properties such as microhardness, wear resistance, surface roughness, or ion release of calcium and phosphate, which may also impact long-term clinical performance. Additionally, the study period of 35 days may not fully capture long-term material behavior and degradation. Finally, experimental conditions such as the use of distilled water rather than artificial saliva or dynamic oral conditions could have influenced the absorption and solubility measurements.

## 5. Conclusions

Based on the results of this study, it can be concluded that exposure to ionizing radiation did not produce statistically significant changes in water absorption or solubility for any of the tested restorative materials across the evaluated time intervals. Notably, Fuji IX, Fuji Triage, Beautifil II, and Equia Forte exhibited higher absolute values of water absorption and solubility relative to the other tested materials, which can be attributed to their hydrophilic composition and matrix characteristics. These findings indicate that, under the applied radiation conditions, the intrinsic physicochemical stability of the materials was preserved. The combination of dimensional stability and bioactive ion release supports both restorative longevity and anti-cariogenic efficacy. Clinicians should therefore consider not only surface characteristics but also composition, hydrophilicity, and filler content when selecting restoratives for high-risk patients. Further longitudinal and in vivo studies are warranted to confirm these in vitro findings, to explore how surface-level radiation effects interact with material properties, and to optimize preventive dental strategies in the context of radiotherapy.

## Figures and Tables

**Table 1:** Compositions and relevant characteristics of the tested materials.

Material	Manufacturer (Country)	Classification/Type	Main Ingredients	Supplier/Purchase Link	LOT
Equia Forte HT	GC Corporation (Tokyo, Japan)	Resin-coated high-viscosity glass ionomer restorative	Powder: fluoroaluminosilicate glass; Liquid: polyacrylic acid solution; Protective light-cured resin coat	https://www.gc.dental	2303108
Cention Forte	Ivoclar Vivadent (Schaan, Liechtenstein)	Alkasite restorative material	Resin matrix (UDMA, DCP, aromatic aliphatic UDMA); alkaline glass fillers (fluoride, calcium, hydroxide release)	https://www.ivoclar.com	ZL08SP
Fuji IX GP Extra	GC Corporation (Tokyo, Japan)	Conventional high-viscosity GIC	Powder: fluoroaluminosilicate glass; Liquid: polyacrylic acid solution	https://www.gc.dental	230105A
Fuji Triage	GC Corporation (Tokyo, Japan)	Glass ionomer-based fissure sealant/protective coating	Powder: fluoroaluminosilicate glass, pigments; Liquid: polyacrylic acid solution	https://www.gc.dental	2206031
Activa Presto	Pulpdent Corporation (Watertown, MA, USA)	Bioactive resin-modified restorative	Ionic resin system; bioactive glass fillers releasing fluoride, calcium, phosphate	https://www.pulpdent.com	220419
Luminos	Unodent (Witham, UK)	Resin-based fluoride-releasing restorative	Methacrylate-based resin matrix; fluoride-containing fillers	https://www.presidentdental.de	20250225
Beautifil II	Shofu Inc. (Kyoto, Japan)	Giomer composite	Resin matrix (Bis-GMA, TEGDMA); surface prereacted glass-ionomer (PRG) fillers	https://www.shofu.com	032266

**Table 2:** Presentation of the absorption by day, material and group.

	Absorption Day 1			Absorption Day 7			Absorption Day 14			Absorption Day 21			Absorption Day 35				Solubility 1		
Material	Type	Median	IQR	*p* Value	Median	IQR	*p* Value	Median	IQR	*p* Value	Median	IQR	*p* Value	Median	IQR	*p* Value	Median	IQR	*p* Value
Activa presto	E	0.0017	0.0015–0.0018		0.0021	0.0019–0.0025		0.00205	0.002–0.0024		0.00525	0.0043–0.0058		0.00505	0.0042–0.0059		0.0099	0.0098–0.016	
Activa presto	C	0.0016	0.0014–0.0018	0.529	0.00205	0.0019–0.0023	0.481	0.00205	0.0019–0.0023	0.436	0.00505	0.0043–0.0058	0.796	0.00485	0.004–0.0058	0.739	0.0098	0.0095–0.014	0.481
Luminos	E	0.0027	0.0025–0.0028		0.0035	0.003–0.0038		0.0032	0.003–0.0034		0.0057	0.005–0.0062		0.0057	0.0052–0.006		0.00875	0.0085–0.0114	
Luminos	C	0.00255	0.0025–0.0026	0.315	0.003	0.003–0.0034	0.28	0.003	0.003–0.0032	0.353	0.0056	0.005–0.0062	0.739	0.0056	0.005–0.0062	0.631	0.0085	0.0083–0.0112	0.481
Beautifil	E	0.0025	0.002–0.003		0.0047	0.0042–0.0052		0.0048	0.004–0.005		0.02115	0.017–0.0234		0.02105	0.017–0.0231		0.01175	0.0013–0.0126	
Beautifil	C	0.00215	0.002–0.0029	0.28	0.0046	0.0042–0.0052	0.579	0.00465	0.004–0.005	0.739	0.02105	0.017–0.0234	0.684	0.02105	0.017–0.023	0.796	0.01175	0.0012–0.0126	0.579
Cention	E	0.00195	0.0018–0.0024		0.00275	0.0025–0.003		0.0029	0.0027–0.0035		0.00855	0.007–0.0096		0.0087	0.007–0.0098		0.00825	0.007–0.0094	
Cention	C	0.0019	0.0018–0.0022	0.684	0.0027	0.0025–0.0028	0.481	0.0028	0.0026–0.0033	0.631	0.0085	0.0069–0.0092	0.579	0.0085	0.0069–0.0092	0.631	0.0086	0.0092–0.664	0.664
Fuji IX	E	0.00295	0.0024–0.0032		0.00385	0.0035–0.0042		0.004	0.0039–0.0046		0.03075	0.0299–0.0317		0.03075	0.0299–0.0317		0.02185	0.0199–0.0235	
Fuji IX	C	0.00285	0.0022–0.003	0.739	0.0037	0.0034–0.004	0.579	0.004	0.0037–0.0045	0.796	0.0303	0.0299–0.0311	0.631	0.0303	0.0299–0.0311	0.353	0.0206	0.0218–0.028	0.28
Fuji Triage	E	0.0033	0.0028–0.0037		0.0054	0.005–0.0057		0.0051	0.0049–0.0054		0.03255	0.032–0.0329		0.03265	0.032–0.0329		0.0221	0.0219–0.0235	
Fuji Triage	C	0.003	0.0027–0.0032	0.579	0.00525	0.005–0.0055	0.315	0.00505	0.0046–0.0052	0.684	0.03235	0.032–0.0329	0.739	0.03235	0.032–0.0329	0.739	0.022	0.0214–0.0232	0.436
Equia	E	0.00235	0.0018–0.0028		0.0027	0.0022–0.0029		0.00285	0.0026–0.003		0.01185	0.0112–0.0123		0.01185	0.0112–0.0123		0.01245	0.0118–0.0129	
Equia	C	0.0022	0.0019–0.0027	0.529	0.0026	0.0024–0.0028	0.796	0.0028	0.0024–0.0032	0.739	0.01165	0.0111–0.0134	0.684	0.01165	0.0111–0.0134	0.684	0.0127	0.012–0.0129	0.684

## Data Availability

The original contributions presented in the study are included in the article, further inquiries can be directed to the corresponding author.
